# A Label-Free Electrochemical Aptamer Sensor for Sensitive Detection of Cardiac Troponin I Based on AuNPs/PB/PS/GCE

**DOI:** 10.3390/nano14191579

**Published:** 2024-09-30

**Authors:** Liying Jiang, Dongyang Li, Mingxing Su, Yirong Qiu, Fenghua Chen, Xiaomei Qin, Lan Wang, Yanghai Gui, Jianbo Zhao, Huishi Guo, Xiaoyun Qin, Zhen Zhang

**Affiliations:** 1 School of Electrical and Information Engineering, Zhengzhou University of Light Industry, Zhengzhou 450000, China; jiangliying@zzuli.edu.cn (L.J.); 332201060098@zzuli.edu.cn (D.L.); 2 School of Material and Chemical Engineering, Zhengzhou University of Light Industry, Zhengzhou 450000, China; 542204040216@zzuli.edu.cn (M.S.); 542104040314@zzuli.edu.cn (Y.Q.); phenix@zzuli.edu.cn (F.C.); qxmqin@zzuli.edu.cn (X.Q.); wanglan@zzuli.edu.cn (L.W.); yhgui@zzuli.edu.cn (Y.G.); zhaojianbo@zzuli.edu.cn (J.Z.); guohuishi@zzuli.edu.cn (H.G.); 3 Tianjin Key Laboratory of Molecular Optoelectronic Sciences, Department of Chemistry, School of Science, Tianjin University, Tianjin 300072, China

**Keywords:** electrochemical sensor, cardiac troponin I, aptamer, Prussian blue, gold nanoparticles

## Abstract

Cardiac troponin I (cTnI) monitoring is of great value in the clinical diagnosis of acute myocardial infarction (AMI). In this paper, a highly sensitive electrochemical aptamer sensor using polystyrene (PS) microspheres as the electrode substrate material in combination with Prussian blue (PB) and gold nanoparticles (AuNPs) was demonstrated for the sensitive and label-free determination of cTnI. PS microspheres were synthesized by emulsion polymerization and then dropped onto the glassy carbon electrode (GCE); PB and AuNPs were electrodeposited on the electrode in corresponding electrolyte solutions step by step. The PS microsphere substrate provided a large surface area for the loading mass of the biological affinity aptamers, while the PB layer improved the electrical conductivity of the modified electrode, and the electroactive AuNPs exhibited excellent catalytic performance for the subsequent electrochemical measurements. In view of the above mentioned AuNPs/PB/PS/GCE sensing platform, the fabricated label-free electrochemical aptamer sensor exhibited a wide detection range of 10 fg/mL~1.0 μg/mL and a low detection limit of 2.03 fg/mL under the optimal conditions. Furthermore, this biosensor provided an effective detection platform for the analysis of cTnI in serum samples. The introduction of this sensitive electrochemical aptamer sensor provides a reference for clinically sensitive detection of cTnI.

## 1. Introduction

Cardiovascular disease (CVD) is the leading cause of death worldwide, accounting for approximately 31% of all deaths, and includes angina, acute myocardial infarction (AMI), unstable angina, and heart failure [1,2]. In particular, AMI is believed to be the leading cause of death in people with cardiovascular disease [3]. The “golden” window for thrombolysis and interventional therapy is 1~3 h after the onset of AMI, which means that rapid diagnosis of early AMI is key to treatment [4]. Traditional diagnosis of AMI is based on angina symptoms, electrocardiography, and biomarker testing [5], with biomarkers being particularly important in identifying patients with atypical presentation (i.e., no chest pain or no ST-segment elevation on electrocardiography) [6]. A number of indicators, including myoglobin, c-reactive protein, lactate dehydrogenase, and cardiac troponin I (cTnI), have proved to be useful in the assessment of AMI [7,8]. Among various biomarkers, cTnI is considered the “gold standard” for early diagnosis of AMI because of its high clinical sensitivity and specificity for cardiac tissue, as well as its ability to reflect small areas of myocardial necrosis or ischemia [9,10,11]. When an AMI occurs, serum cTnI concentrations rise within 12 h and remain elevated for 5~9 days [12]. In healthy individuals, cTnI concentrations are typically below 0.4 ng/mL, while levels above 2.0 ng/mL are associated with an increased risk of potential future major cardiac events [9].

Numerous techniques, including fluorescent [13], colorimetric [14], and electrochemical [15], have been developed for the detection of cTnI. One of the most commonly used clinical techniques is the enzyme-linked immunosorbent assay (ELISA), which is specific and sensitive but has a long detection time and results are affected by many factors [16]. While electrochemical methods have the advantages of rapid response, low cost, time savings, and high sensitivity [15]. Most electrochemical methods for cTnI detection are based on antibody-antigen interactions but suffer from poor stability, low robustness, and high cost. Dorraj et al. [17] screened four DNA aptamers against the cTnI protein using a systematic evolution of ligands by exponential enrichment (SELEX) method, which have high binding affinities with dissociation constants (*K*_d_) in the nanomolar range. Jo et al. [8] further selected the Tro4 aptamer for cTnI with a very low *K*_d_ value (270 pM) compared to that of a cTnI antibody (20.8 nM). Aptamers can be used as an alternative to antibodies in the determination of cTnI because they overcome the limitations of antibodies, eliminate the ethical concerns associated with animal or human products, ensure no batch-to-batch variation, and allow selection under non-physiological conditions [18,19].

Prussian blue (PB, ferric hexacyanoferrate) is widely used as a signal indicator due to its easy synthesis, low cost, no electroactive molecules kinetic diffusion, and high physical stability. In addition, as a metal complex, PB has higher chemical stability and can ensure the continuous output of the signal, thereby improving the durability and reproducibility of the sensor. PB is typically chemically synthesized in a conventional manufacturing process by a mixed reaction of ferric and hexacyanoferrates with different oxidation states of iron ions [20]. The reaction proceeds too fast, resulting in an inability to regulate the size and shape of the formed PB crystals to guarantee high reproducibility [21]. It has been found that some carbon nanomaterials, such as carbon nanotubes [22], graphene [23], and carbon nanospheres [24], have proven to be effective platforms for assisting PB synthesis. The present work attempts to explore polystyrene (PS) microspheres as a substrate to support the electrodeposition of PB, as well as to provide a large surface area to overcome aggregation and improve biopolymer stabilization. In order to enhance the catalytic ability and amplify the electrochemical signals, gold nanoparticles (AuNPs) are widely used in the construction of electrochemical biosensors due to their good biocompatibility and ease of functionalization [25,26]. Various bioreceptors, such as DNA, enzymes, and antibodies, could be attached to the surface of gold nanomaterials through an Au-S bond or chelation with amino and carboxyl groups. Zhang et al. [27] used PS microspheres as signal carriers to construct a brush structure by conjugating gold nanorods, and the designed nanostructure greatly increased the amount of loaded capture antibodies. Sun et al. [28] exploited chitosan-functionalized graphene oxide composites to provide abundant active sites for anchoring AuNPs and subsequently immobilized cTnI antigen through Au-N bonds. However, the complicated structural design and multi-step synthesis procedure require skilled experimental techniques, which raises the threshold for the preparation of modified electrodes and makes it difficult to prepare large-scale batch-level sensors with high reproducibility. For the fabrication of modified electrodes with high reproducibility, electrodeposition offers a practical alternative as a standardized preparation method.

**Scheme 1:**
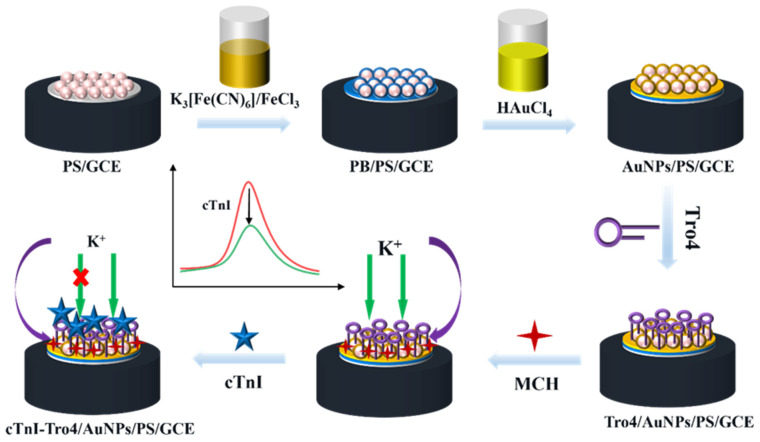
Schematic diagram of the construction procedure of the Tro4/AuNPs/PB/PS/GCE aptamer sensor and its electrochemical sensing for cTnI.

Herein, we developed a label-free electrochemical aptamer sensor for the sensitive detection of cTnI based on AuNPs/PB/PS modified glassy carbon electrode (GCE), where PB and AuNPs were successively electrodeposited on the PS microspheres modified GCE (Scheme 1). The thiol-functionalized aptamer Tro4 was chemically linked to the AuNPs/PB/PS/GCE through the Au-S bond. The constructed electrochemical aptamer sensor was then subjected to the sensitive detection of cTnI with a wide detection range of 10 fg/mL~1.0 μg/mL and a low detection limit of 2.03 fg/mL under the optimal conditions. Furthermore, the reliable anti-interference and detectability in serum samples were confirmed, indicating its promising application in the early diagnosis of cardiovascular diseases.

## 2. Experimental Section

### 2.1. Chemicals and Apparatus

Cardiac troponin I (cTnI), cardiac troponin T (cTnT), hemoglobin (Hb), and myoglobin (Myo) were purchased from Wuhan Yunclone Technology Co., Ltd. (Wuhan, China). The aptamer (Tro4, SH-(C_6_)-5′-CGTGCAGTACGCCAACCTTTCTCATGCGCTGCCCCCCTCTTA) was purchased from Shanghai Sangong Bioengineering Co., Ltd. (Shanghai, China). Tris (hydroxymethyl) aminomethane (Tris-HCl) was supplied by Sinopharm Chemical Reagent Co., Ltd. (Shanghai, China). Styrene (C_8_H_8_) was purchased from Shanghai McLean Biochemistry and Technology Co., Ltd. (Shanghai, China). Chloroauric acid (HAuCl_4_), 6-mercaptohexanol (MCH), sodium hydroxide (NaOH), polyvinylpyrrolidone (PVP), K_3_[Fe(CN)_6_], FeCl_3_∙6H_2_O, KCl, KNO_3_, HCl, ethanol, and MgCl_2_ were purchased from Shanghai Aladdin Biochemical Technology Co., Ltd. (Shanghai, China). Ultrapure water with a resistivity > 18 ΩM was provided by an ultrapure water machine from ELGA Biochemistry (High Wycombe, UK). All the chemical reagents used in this experiment were analytically pure and were used as received. All data in the paper were measured three times to obtain error bars.

All electrochemical measurements were performed on a CH Instruments model 760E electrochemical analyzer (CH Instruments, Inc., Shanghai, China). The traditional three-electrode system was used for electrochemical detection: GCE with a diameter of 6 mm was used as the working electrode, the reference electrode was Ag/AgCl, and the auxiliary electrode was a platinum column plate. The electrochemical measurement methods used included cyclic voltammetry (CV), electrochemical impedance spectroscopy (EIS), and differential pulse voltammetry (DPV). The electrochemical measurements of CV were performed in 0.1 M PBS containing 5 mM [Fe(CN)_6_]^3−/4−^ solution (pH = 7.4). A potential range of −0.2~0.6 V was selected for the measurements to observe the redox peaks, with a step potential of 0.01 V and a potential scanning rate of 100 mV/s. EIS was performed in 0.1 M PBS with 5 mM [Fe(CN)_6_]^3−/4−^ (pH = 7.4), with a frequency range of 0.1 Hz to 100 kHz, an amplitude of 5 mV, and an open-circuit potential of −0.2 V.

The morphology of the modified electrodes was characterized by scanning electron microscopy (SEM, JSM-60, JEOL, Tokyo, Japan) operating at an accelerating voltage of 20 kV. Energy dispersive X-ray analysis (EDAX) was performed using a JSM-60 (JEOL, Tokyo, Japan) equipped with an Oxford Extreme windowless EDX detector (Oxford Instruments, High Wycombe, UK). XRD was performed on a Rigaku Dmax-2500 X-ray diffractometer (Rigaku, Tokyo, Japan) using Cu Ka radiation (λ = 1.54 Å) at 50 kV and 200 mA at a scan rate of 5 °/min. Fourier transform infrared (FT-IR) spectra were obtained using a Fourier transform infrared spectrometer (vertex70, Brock Instruments, Bochum, Germany).

### 2.2. Preparation of PS Microspheres

PS microspheres were synthesized by emulsion polymerization method. In a three-necked flask, 80 g of ethanol was added, and then 1.44 g of PVP was added. After the PVP was completely dissolved, the temperature was raised to 70 °C, and 15 g of styrene monomer was added. After stirring for 10 min to disperse the styrene uniformly, the initiator azobisisobutyronitrile (AIBN) was added to initiate the polymerization of the styrene monomer, and the temperature was kept constant for 10 h under a nitrogen atmosphere. The prepared PS solution was centrifuged (8000 rpm) for 5 min to remove the float, and the resulting precipitate was added to 50 mL of ethanol to redisperse by ultrasonic treatment. The precipitates were collected, weighed, and dispersed in ethanol at a mass concentration of 1 mg/mL.

### 2.3. Preparation of AuNPs/PB/PS/GCE

Prior to modification, the bare GCE was successively polished with 1.0 μm, 0.3 μm, and 0.05 μm Al_2_O_3_ powder. It was then subjected to 15 CV scans in 0.5 M H_2_SO_4_ with a potential window of −0.5~1.8 V at a scan rate of 100 mV/s to activate the electrode. Finally, the electrode surface was dried with an N_2_ blow dryer. To obtain PS/GCE, 5 μL of PS solution was applied dropwise to the GCE, dried in a desiccator, and then the surface was cleaned with ultrapure water. The PS/GCE was immersed in a mixed solution consisting of 1 mM K_3_[Fe(CN)_6_], 1 mM FeCl_3_∙6H_2_O, 0.1 M KCl, 0.92 mM CTAB, and 0.02 mM HCl. The PB film was electrodeposited by conducting CV scanning for 15 runs in the potential range of 0~1.0 V at a rate of 50 mV/s. The obtained PB/PS/GCE was then placed in 1 mM HAuCl_4_ solution and subjected to 20 runs of CV cycles scanning from −0.5 V to 0 V for the electrodeposition of AuNPs on the electrode at a scanning rate of 100 mV/s. The electrode was rinsed with ultrapure water to obtain AuNPs/PB/PS/GCE.

### 2.4. Preparation of Tro4/AuNPs/PB/PS/GCE

The AuNPs/PB/PS/GCE were immersed in a buffer solution (20 mM Tris-HCl, 5 mM MgCl_2_, and 50 mM NaCl, pH = 7.4) containing 1 µM Tro4 and incubated at 4 °C for 6 h to generate Tro4/AuNPs/PB/GCE. The modified electrode was then washed with 20 mM Tris-HCl buffer to remove the unbound aptamer, and then 10 μL of 1 mM MCH solution was added to the surface of the Tro4/AuNPs/PB/GCE to block the non-specific active site on the electrode surface.

### 2.5. Electrochemical Detection of cTnI

In this experiment, an immersion method was used to investigate the sensing performance of the Tro4/AuNPs/PB/PS/GCE aptamer sensor in this experiment for the target cTnI. The constructed sensors were immersed in various cTnI concentrations ranging from 10 fg/mL to 1.0 μg/mL and incubated at 37 °C for 1 h. Then, the electrodes were washed with deionized water, and the electrochemical signals were recorded using DPV analysis mode in a 0.8 M KNO_3_ solution with a potential range of −0.2 to 0.6 V, a modulation amplitude of 50 mV, a step potential of 4 mV, an experimental pulse period of 0.5 s, and an experimental pulse width of 0.05 s.

### 2.6. Measurement of Human Serum Samples

To further investigate the ability of the aptamer sensor to analyze real samples, we used the standard addition method in serum dilutions to further investigate the sensing performance, as the standard addition procedure eliminates the matrix effect in serum samples. Human serum albumin samples were purchased from Beijing Solarbio Life Sciences Ltd. (Beijing, China). 20 mM Tris-HCl and 150 mM NaCl (pH 7.4) were used to dilute the serum to 10% as a buffer for the detection of cTnI. Various concentrations of cTnI were then spiked to the serum dilution to simulate healthy individuals (60 fg/mL, 0.3 pg/mL, 1 pg/mL) and AMI patients (for mild ones, 15 pg/mL, 30 pg/mL, 40 pg/mL, 40 pg/mL, 60 pg/mL, 80 pg/mL; for severe ones, 1.5 ng/mL, 10 ng/mL, 35 ng/mL, 150 ng/mL). The constructed Tro4/AuNPs/PB/PS/GCE aptamer sensors were immersed in the spiked serum dilution and incubated at 37 °C for 1 h. The electrodes were washed with deionized water, and the electrochemical signals were recorded using the DPV method as described above.

## 3. Results and Discussion

### 3.1. Characterization of AuNPs/PB/PS/GCE

**Figure 1:**
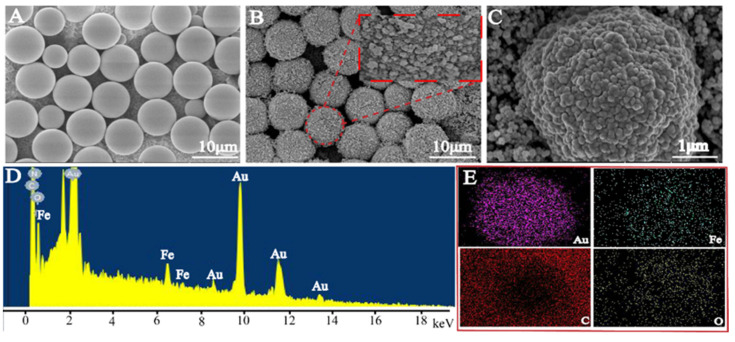
SEM images of (**A**) PS, (**B**) PB/PS, and (**C**) AuNPs/PB/PS microspheres. (**D**) EDAX spectrum of AuNPs/PB/PS. (**E**) The mapping images of a single sphere for AuNPs/PB/PS, (magenta) Au, (green) Fe, (red) C, and (yellow) O elemental spectra.

SEM and EDAX were employed to investigate the morphology and elemental distribution of the modified electrodes. Figure 1A exhibited the smooth PS microspheres with diameters ranging from 4 to 9 μm. Figure 1B displayed the morphology of the PS-modified electrode after an electrodeposition treatment in a mixed solution of ferric chloride and potassium ferricyanide, and the localized zoomed image shown in the red dashed box indicated that the smooth surface of the PS microsphere was uniformly covered with a layer of rough PB nanoparticles. The EDAX spectrum and elemental mapping images further indicated the successful fabrication of PB/PS as shown in Figure S1, where the Fe element was adhered to the sphere surface. The morphology obtained by further electrodeposition of AuNPs on PB/PS/GCE was shown in Figure 1C, where denser nanoparticles covering the microsphere surface can be observed. Due to the better conductivity of the AuNPs, the obtained AuNPs/PB/PS product has better contrast, resulting in a sharper image. Figure 1D exhibited the EDAX spectrum of AuNPs/PB/PS, indicating the presence of C, N, O, Fe, and Au elements. The elemental contents for the obtained AuNPs/PB/PS were listed as C 79.98 at%, N 10.23 at%, O 5.74 at%, Fe 0.28 at%, and Au 3.77 at% (Table S1). The elemental mapping images for Au, Fe, C, and O were collected as shown in Figure 1E, from which Au can be found on the surface of the sphere, further confirming the successful preparation of the AuNPs/PB/PS nanostructure.

**Figure 2:**
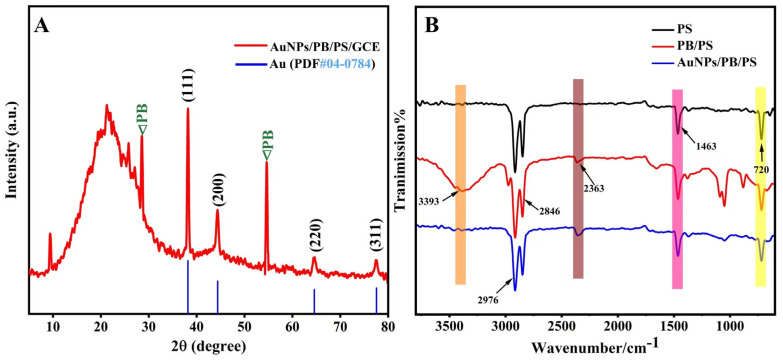
(**A**) XRD pattern of AuNPs/PB/PS (red curve). (**B**) FT-IR spectra of PS (black curve), PB/PS (red curve), and AuNPs/PB/PS (blue curve).

The XRD pattern and FT-IR spectra of the modified electrode are shown in Figure 2 to characterize the structure and surface chemical state. As shown in Figure 2A, the presence of PS microspheres resulted in a strong and broad background peak around ~21° in the XRD pattern of AuNPs/PB/PS, which was attributed to the amorphous carbon structure. It can be observed that the diffraction peaks at 38°, 44°, 65°, and 78° can be assigned to (111), (200), (220), and (311) faces of metallic Au (PDF#04-0784). The peaks around 28° and 55° were assigned to PB [29]. The FT-IR spectra of PS, PB/PS, and AuNPs/PB/PS are shown in Figure 2B. The absorption peaks at 2976 cm^−1^, 2846 cm^−1^, and 1463 cm^−1^ can be attributed to the asymmetric, symmetric stretching vibration, and bending vibrations of C-H in the PS, respectively. The moderate peak at 720 cm^−1^ was a typical out-of-plane bending vibration of C–H in the –(CH_2_)*_n_*– chain in PS. Compared with PS, PB/PS and AuNPs/PB/PS exhibited an additional absorption peak at 2363 cm^−1^, which can be attributed to the C≡N stretching vibration in the formed [Fe(II)-CN-Fe(III)] structure, indicating the successful electrodeposition of PB on the PS surface. The broad and moderately intense absorption peak at ~3393 cm^−1^ can be attributed to the –NH stretching vibration, which disappeared after the deposition of AuNPs, suggesting that the additional AuNPs can be anchored on the PB/PS surface by the bonding of Au with –NH leading to the disappearance of this peak. Based on the XRD and FT-IR results, it was shown that both PB and AuNPs were successfully electrodeposited on the PS surface.

### 3.2. Characterization of the Electrochemical Aptamer Sensor

**Figure 3:**
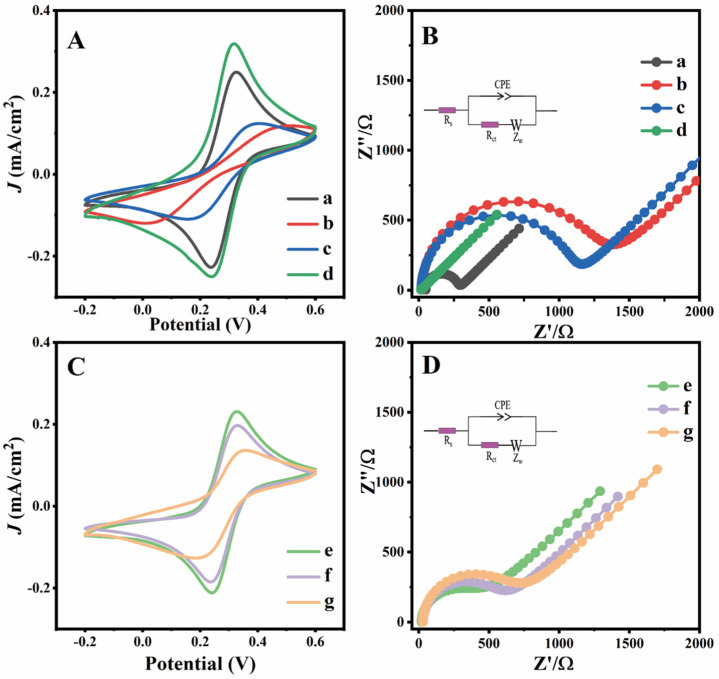
(**A**,**C**) CV curves and (**B**,**D**) EIS plots of (a) GCE, (b) PS/GCE, (c) PB/PS/GCE, and (d) AuNPs/PB/PS/GCE. (**C**) CV curves and (**D**) EIS plots of (e) Tro4/AuNPs/PB/PS/GCE, (f) MCH/Tro4/AuNPs/PB/PS/GCE, and (g) cTnI/MCH/Tro4/AuNPs/PB/PS/GCE. Dotted line, raw data; solid line, fitted result.

As shown in Figure 3, electrochemical measurements using CV and EIS techniques were performed on bare and modified GCEs in 0.1 M PBS solution containing 5 mM [Fe(CN)_6_]^3−/4−^ as a redox-active couple to evaluate the modification processes of the modified electrodes. It can be observed that after the modification of PS microspheres (curve b), the redox peak current was significantly decreased compared to the bare GCE (curve a) due to the poor conductivity of PS to hinder the charge transfer between the redox ions and the electrode (Figure 3A). The electrodeposition of the PB layer on PS/GCE (curve c) resulted in an increase in the peak current because of the good electrical conductivity of PB. Further electrodeposition of AuNPs (curve d) greatly increased the peak current density (*J*) from 0.12 mA/cm^2^ (PB/PS/GCE) to 0.31 mA/cm^2^ (AuNPs/PB/PS/GCE), indicating that the prepared modified electrode has a sensitive response due to the excellent conductivity of AuNPs and a more continuous microsurface to ensure the unobstructed electron transport path. The surface of the AuNPs/PB/PS/GCE was immobilized with the 5′-thiol-Tro4 aptamer probes (curve e) through Au-S bonds and subsequently blocked with MCH (curve f) to prevent non-specific binding (Figure 3C). The insulating organic matter would impede electron transfer and cause a current density decrease in the redox peaks, and the sealer MCH further decreased the peak current. The feasibility of the electrochemical aptamer sensor was demonstrated by incubating the MCH/Tro4/AuNPs/PB/PS/GCE with the target for 1 h. In the presence of cTnI (curve g), Tro4 is specifically bound to the protein molecule, preventing the access of [Fe(CN)_6_]^3−^/[Fe(CN)_6_]^4−^ to the electrode surface. Consequently, a decrease in the redox peak current density was observed, reflecting the presence of the target cTnI. Therefore, the designed aptamer sensor is capable of highly sensitive electrochemical detection of cTnI.

EIS serves as a common technique for evaluating electron transfer capability. Nyquist plots accompanied by a Randles model equivalent circuit are shown in the inset in Figure 3B,D, where *R*_ct_, *R*_s_, *C*_dl_, and *Z*_W_ represent the charge transfer resistance, solution resistance, double-layer capacitance, and the Warburg resistance, respectively. The corresponding EIS results fitted by the equivalent circuit are given in Table S2. In the Nyquist plot, the diameter of the semicircular portion appearing in the high-frequency region indicates the electron-transfer-limited process, the diameter of which is equal to the charge-transfer resistance. First, the EIS of the bare GCE (curve a) was examined, which showed a very small semicircular region with an *R*_ct_ value of 226 ± 7 Ω. After the modification of PS microspheres (curve b), the impedance value increased dramatically to 1202 ± 24 Ω. Further electrodeposition of the PB layer contributed to a lower *R*_ct_ value of 1045 ± 24 Ω (curve c). Moreover, for the deposition of AuNPs, the impedance spectra of AuNPs/PB/PS/GCE (curve d) showed almost a line with an *R*_ct_ value of 13 ± 4 Ω, indicating that AuNPs can greatly enhance the electron transfer rate between the electrode and [Fe(CN)_6_]^3−/4−^ solution. The introduction of AuNPs also provided efficient anchoring sites for the aptamer to capture the target protein. Subsequently, a self-assembled monolayer of thiol-terminated Tro4 was immobilized on the AuNPs/PB/PS/GCE surface (curve e), and as a result, the semicircle diameter increased significantly to 470 ± 27 Ω. When MCH (curve f) was sequentially modified on the electrode, the non-specific binding sites were blocked to further increase the semicircle diameter with an *R*_ct_ value of 514 ± 24 Ω. After the recognition reaction of the MCH/Tro4/AuNPs/PB/PS/GCE sensor with 100 ng/mL cTnI for 1 h (curve g), the impedance increased significantly to 606 ± 26 Ω due to the formation of the immune complex [30]. Accordingly, these impedimetric results were in agreement with the voltammetric results.

**Figure 4:**
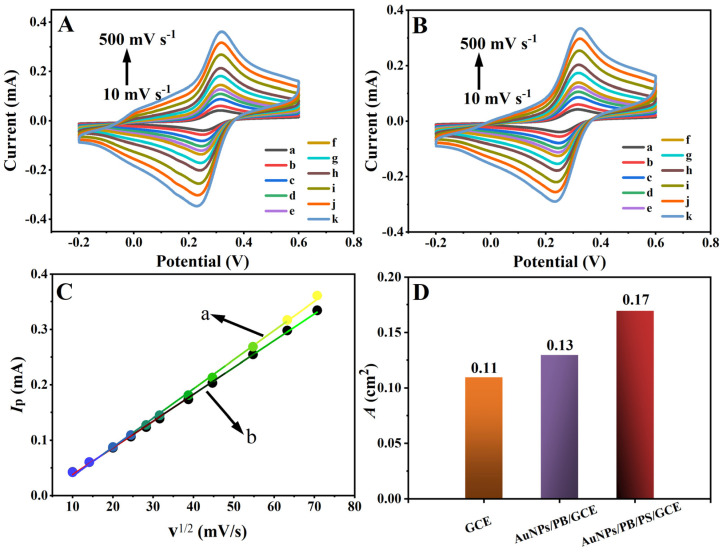
CV plots of (**A**) AuNPs/PB/PS/GCE and (**B**) AuNPs/PB/GCE at different sweep rates. From inside to outside: 10 (curve a), 20 (curve b), 40 (curve c), 60 (curve d), 80 (curve e), 100 (curve f), 150 (curve g), 200 (curve h), 300 (curve i), 400 (curve j), and 500 mV/s (curve k). (**C**) Plot of the oxidation peak current versus the square root of the sweep rate (ν^1/2^) for the (a) AuNPs/PB/PS/GCE and (b) AuNPs/PB/GCE. (**D**) A histogram comparing the corresponding electrochemically active areas of GCE (yellow column), AuNPs/PB/GCE (violet column), and AuNPs/PB/PS/GCE (red column).

In order to investigate the electrochemical active area of the modified electrodes, we performed the CV measurements on Au/PB/PS/GCE and Au/PB/GCE electrodes in 0.1 M PBS containing 5 mM [Fe(CN)_6_]^3−/4−^ with increasing sweep rates from 10 mV/s to 500 mV/s (Figure 4A,B). As the sweep rate increased, the peak redox current signal increased as well. The data presented in Figure 4C indicated that the oxidation peak current Ip was directly proportional to the square root of the scanning speed (*v*^1/2^). This suggested that a diffusion-controlled process existed in both the Au/PB/PS/GCE and Au/PB/GCE electrodes. The Randles-Sevcik equation can be used to determine the electrochemically active area of the constructed electrode [31]:

*I*_p_ = 2.69 × 10^5^*n*^3/2^*AD*^1/2^*ν*^1/2^*C* (1)

where *I*_p_ is the peak current (A), *n* is the number of electrons transferred (*n* = 1), *A* is the electrochemically active area of the electrode (cm^2^), *D* is the diffusion coefficient of [Fe(CN)_6_]^3−/4−^ (7.6 × 10^−6^ cm^2^/s), *C* is the concentration of the reactant (5 × 10^−6^ mol/cm^3^), and *ν* is the sweep rate (V/s). As shown in Figure S2, the current response of the bare GCE electrode was the smallest and became larger after the modification of PB and AuNPs due to their excellent conductivity. In Figure 4D, the electrochemically active areas were compared in a histogram for bare GCE, AuNPs/PB/GCE, and AuNPs/PB/PS/GCE, from which we could find that AuNPs/PB/PS/GCE had the best performance. The AuNPs/PB/GCE exhibited a higher electrochemically active area of 0.13 cm^2^ than that of bare GCE of 0.11 cm^2^. Additional PS microspheres further introduced a larger electrochemically active area of 0.17 cm^2^, suggesting that the introduction of PS microspheres in the modified electrode would increase the surface roughness and provide a larger surface area.

### 3.3. Optimization of Experimental Conditions

The electrodeposition cycles for PB and AuNPs on the PS/GCE were investigated to optimize the preparation parameters. Figure S3 showed the CV plot for a cycle number of 25 turns in the electrodeposition of PB, illustrating the good redox peak signals of the PB electrodeposited on the electrode surface. As the number of cycles increased, the redox peak current density also increased. The responses that occur during the process are,

4FeCl_3_ + 3K_4_[Fe(CN)_6_] → Fe_4_[Fe(CN)_6_]_3_ (PB)↓ + 12KCl (2)

The redox peak current density did not increase and maintained a strong electrochemical signal after 15 cycles of scanning. Therefore, 15 cycles of electrodeposition were determined to be the ideal condition for the electrodeposited PB in this experiment. The performance of the modified electrode was also affected by the amount of AuNPs electrodeposited at different cycles, as shown in Figure S4. As the number of electrodeposition cycles increased, the AuNPs on the electrode surface continued to grow, and the redox current density became larger and larger. However, as the number of AuNPs increased until the nanoparticles were clustered together, the specific surface area began to decrease, resulting in a decrease in the active area of the electrode as well. Therefore, the peak current density was highest at 15 cycles and decreased when the number of gold plating cycles was 20 cycles. Therefore, the electrodeposition of 15 cycles was determined to be the ideal condition for electrodeposited AuNPs in this experiment.

In order to obtain the best performance of the electrochemical biosensor, the following experimental conditions were optimized, including the concentration of K^+^, the incubation time of Tro4, MCH, and the target cTnI. As shown in Scheme 1, the sensing mechanism of cTnI was based on an ion barrier effect that the specific binding of cTnI could block the electrode surface and prevent the reaction between K+ and PB to form Prussian white (PW) according to the following reaction:

Fe_4_[Fe(CN)_6_]_3_ (PB) + 4e^−^ + 4K^+^ → K_4_Fe_4_[Fe(CN)_6_]_3_ (PW) (3)

**Figure 5:**
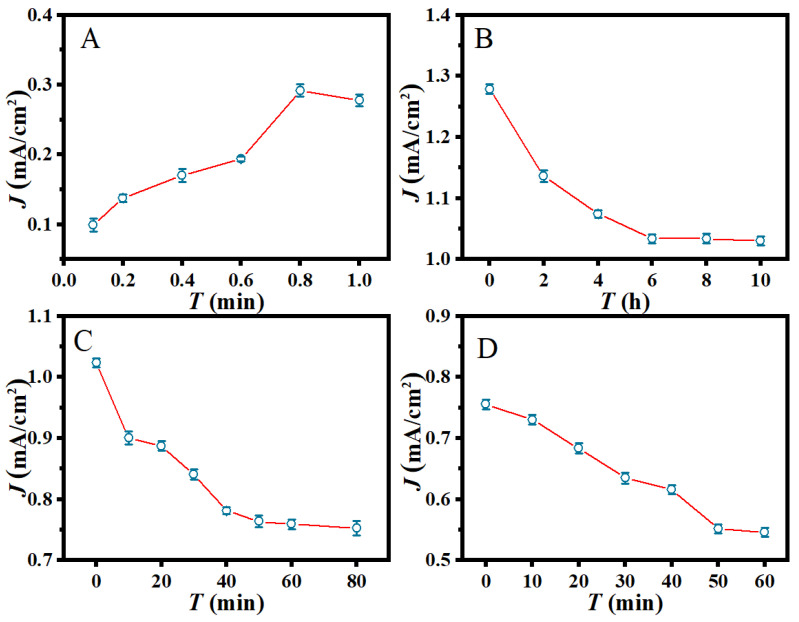
(**A**) Effect of different concentrations of KNO_3_ on the peak current density. (**B**) Effect of different incubation time of Tro4 on the peak current density. (**C**) Effect of different blocking time of MCH on the peak current density. (**D**) Effect of the binding time between Tro4/AuNPs/PB/PS/GCE and cTnI on the peak current density.

Therefore, the concentration of K^+^ should be optimized to achieve a more sensitive detection signal. As shown in Figure 5A, as the concentration of the KNO_3_ solution increased, the redox peak current density increased while the potential difference decreased due to the redox reaction between PB and PW. With the increase in K^+^ concentration, more Fe^3+^ ions participated in the reaction, which then accelerated the chemical reaction rate and improved the redox reversibility. Finally, the current signal increased. According to the Nernst formula,

*E* = *E^θ^* + (0.0592/*n*) ln([Fe^3+^]/[Fe^2+^]) (4)

with the increase in the concentration of K^+^, the concentration of Fe^3+^ involved in the reaction increased, the value of [Fe^3+^]/[Fe^2+^] became larger, and the potential shifted forward so that the potential difference of the redox peak decreased. When the concentration of KNO_3_ was above 0.8 M, the redox peak signal reached the maximum, indicating that the reaction reached the saturation state. The potential difference of the redox peak was less than 60 mV, demonstrating the good redox reversibility of PB. Therefore, a 0.8 M KNO_3_ solution was selected as the detection medium for cTnI. The AuNPs/PB/PS/GCE were immersed in 1 µM Tro4 buffer solution for different periods of time to optimize the Tro4 incubation time. As shown in Figure 5B, the DPV response signal of the modified electrode gradually decreased with increasing immersion time. The peak current density reached a plateau and remained when the incubation time exceeded 6 h. Therefore, 6 h of incubation with Tro4 was selected for the following sensing measurements. Similarly, the incubation time of MCH to block the non-specific active site on the electrode surface was also investigated by immersing Tro4/AuNPs/PB/PS/GCE in 1 mM MCH solution. As shown in Figure 5C, the DPV signals decreased with increasing immersion time. The optimal blocking time for MCH was chosen to be 60 min based on timesaving and acceptable sensitivity. The ideal hybridization time between Tro4/AuNPs/PB/PS/GCE and cTnI was investigated as shown in Figure 5D. The DPV signals decreased continuously with the incubation time of cTnI and remained unchanged after 60 min. Therefore, 60 min of incubation with cTnI was chosen for the following detection measurements.

### 3.4. Detection of cTnI

**Figure 6:**
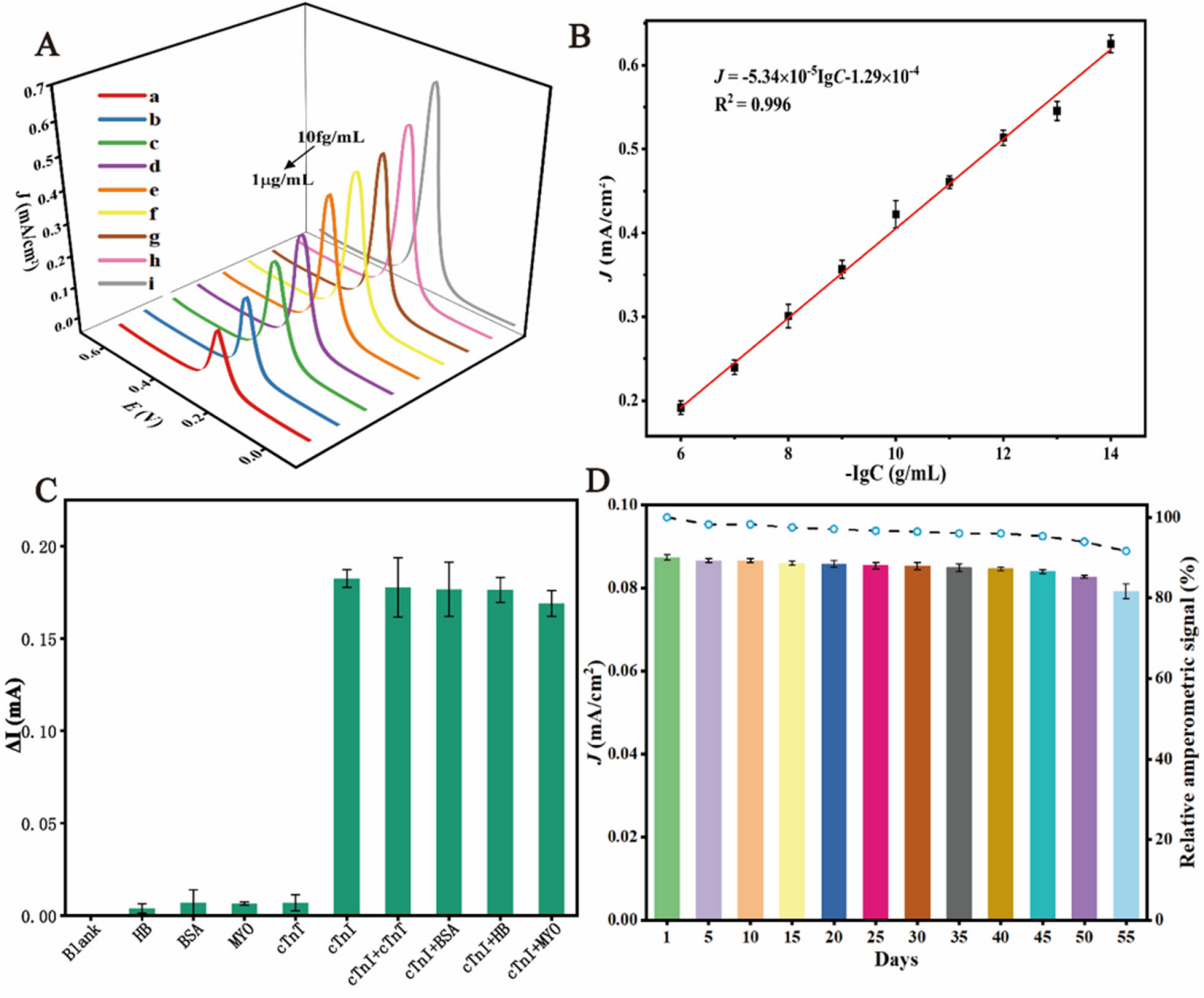
(**A**) Typical DPV current density response of Tro4/AuNPs/PB/PS/GCE to a series of cTnI concentrations in 0.8 M KNO_3_ solution, 10 fg/mL (curve a), 0.1 pg/mL (curve b), 1 pg/mL (curve c), 10 pg/mL (curve d), 0.1 ng/mL (curve e), 1 ng/mL (curve f), 10 ng/mL (curve g), 0.1 μg/mL (curve h), 1.0 μg/mL (curve i). (**B**) The linear correlation between the current density value and the negative logarithm of the cTnI concentration (−lg *C*_[cTnI]_). (**C**) Selectivity and anti-interference performance of the aptamer sensor. (**D**) Stability of the sensor over a period of 55 days; the error bars indicate the standard deviation (*n* = 5).

With the optimized parameters, electrochemical analyses were performed in triplicate for each concentration of cTnI. The constructed Tro4/AuNPs/PB/PS/GCE prehybridized with different concentrations of cTnI were placed in a 0.8 M KNO_3_ solution to detect the electrochemical signal by DPV, as shown in Figure 6A. The peak currents of DPV gradually decreased as the concentration of cTnI increased, which was explained by the fact that the specific binding of Tro4 to cTnI would further inhibit the K^+^ reaction with PB, thereby blocking the reduction of PB (Fe^3+^) to PW (Fe^2+^). The plotted calibration curve shown in Figure 6B exhibited that there was a linear relationship between the peak value of DPV and the negative logarithm of the concentration of the cTnI in the range of 10 fg/mL to 1.0 μg/mL. The linear equation can be written as: *J* = 5.34 × 10^−5^ lg*C* – 1.29 × 10^−4^ (R^2^ = 0.996), and the limit of detection (LOD) was determined to be 2.03 fg/mL (S/N = 3). The analytical performance of the constructed Tro4/AuNPs/PB/PS/GCE sensor was compared with previously reported works in Table 1 [8,18,32,33,34,35,36,37]. It can be concluded that our sensor was easy to prepare and exhibited comparable analytical performance with previously reported electroanalytical methods. To investigate the selectivity of the developed aptamer sensor, a series of proteins such as hemoglobin (HB), bovine serum albumin (BSA), myoglobin (MYO), and cTnT were used for comparison. The concentration of all interferents was chosen as 1 ng/mL, while cTnI was chosen as 0.1 ng/mL. As shown in Figure 6C, the aptamer sensor for interferers showed negligible change in current response compared to cTnI, indicating good selectivity for cTnI detection. Furthermore, the anti-interference performance of the aptamer sensor was also investigated by analyzing Tro4/AuNPs/PB/GCE in different mixed solutions of cTnI (0.1 ng/mL) with other interferents (1 ng/mL). As presented in the plotted histogram in the latter part of Figure 6C, there was no significant change in the current density signal of the sensor after incubation of these mixtures compared to the target cTnI alone, indicating a good anti-interference performance for the determination of cTnI. In addition, the reproducibility of the Tro4/AuNPs/PB/PS/GCE sensor was evaluated for five separately fabricated aptamer sensors over 55 consecutive days. As shown in Figure 6D, intra-assay reproducibility was confirmed by the fact that the five sensors showed almost identical current response to a 10 ng/mL cTnI under the same experimental conditions with a relative standard deviation (RSD) of 3.6%. The sensors were then stored at 4 °C for 55 days, and the DPV responses were collected every five days. The data showed that the current response decreased only slightly after 55 days, and the aptamer sensor retained approximately 92% of the initial current response compared to freshly prepared electrodes, confirming the satisfactory stability of the proposed sensor.

### 3.5. Real Sample Analysis

**Figure 7:**
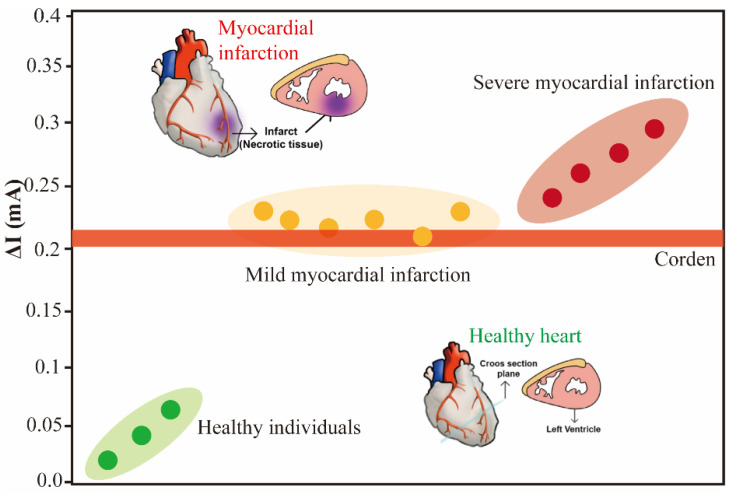
The feasibility of the aptamer sensor in simulated AMI samples.

To validate the practical application of the proposed aptamer sensor for the determination of the cTnI in the real samples, the Tro4/AuNPs/PB/PS/GCE-based sensor system was analyzed in spiked human serum samples by using the standard addition method, which can eliminate the matrix effect [32,34]. Different amounts of cTnI were added to the serum samples to simulate the healthy individuals, mild cases, and severe patients. As shown in Figure 7, there was a clear demarcation of peak current values between patients and normal subjects. The spike recovery assays of the cTnI were also performed using human serum samples diluted 10-fold with 20 mM Tris-HCl (pH 7.4) solution. The results presented in Table 2 demonstrated that the proposed aptamer sensor exhibited excellent capability in determining cTnI in the real samples. Meanwhile, the recovery rates ranged from 101.3% to 104%, and the RSD ranged from 2.7% to 4.7%.

## 4. Conclusions

In conclusion, an aptamer sensor for the detection of cTnI was constructed by a two-step electrodeposition of PB and AuNPs on the PS microspheres modified electrode. The modification layer of PS microspheres could help to homogeneously disperse PB and AuNPs and increase the active area of the electrode to enhance the sensitivity of the sensor. The proposed Tro4/AuNPs/PB/PS/GCE aptamer sensor exhibited a wide linear range of 10 fg/mL~1 μg/mL and a LOD of 2.03 fg/mL under optimized experimental conditions. In addition, the aptamer sensor has high selectivity and anti-interference, good stability, and reproducibility, which could be a promising sensor platform for the detection of cTnI in clinical samples. As a result, the sensor holds great promise to aid in the early detection and prognostic assessment of AMI.

## Figures and Tables

**Table 1:** Comparison of the sensing performance of different biosensors for the detection of cTnI.

Method	Immobilized Receptor	Electrode Material	Linear Range (ng/mL)	LOD (pg/mL)	Ref.
SWV	Tro4	Fc-Si nanoparticles	0.024–240	24	[8]
ELISA	Tro4-Tro6	Fe_3_O_4_-Au-TAPT-TDNs	0.05–100	27	[32]
EIS	Tro4	MoS_2_ nanosheets	0.024–24	23	[33]
*i*-*t*	Tro4-Tro6	HMCS@PDA@AuNPs PtCu DNs/MUN-CuO-TiO_2_	1 × 10^−5^–500	2.3 × 10^−3^	[34]
*i*-*t*	Aptamer 1-Aptamer 2	Au/Zr–C/PtCuNi	1 × 10^−5^–100	1.24 × 10^−3^	[35]
DPV	Tro6	PDA-Au-Pb^2+^/Cu^2+^/Tro6	1 × 10^−5^–1000	3.8 × 10^−3^	[18]
DPV	thiol-aptamer	AuNPs/Ti_3_C_2_-MXene	2.424 × 10^−4^–3	0.14 × 10^−3^	[36]
DPV	Fc-COFNs aptamer	CSA/MCH/Fc-COFNs-MBA/AuE	1 × 10^−5^–10	2.6 × 10^−3^	[37]
DPV	Tro4	Tro4/AuNPs/PB/PS/GCE	1 × 10^−5^–1000	2.03 × 10^−3^	This work

**Table 2:** cTnI recovery assay in diluted human serum samples.

Samples No.	Added (ng/mL)	Measured (ng/mL)	Recovery Rate (%, *n* = 3)	RSD (%, *n* = 3)
1	0.1	0.10	102.0%	2.7%
2	1	1.04	104.0%	4.7%
3	10	10.13	101.3%	4.5%

## Data Availability

Data is contained within the article or Supplementary Materials.

## Supplementary Materials

The following supporting information can be downloaded at: https://www.mdpi.com/article/10.3390/nano14191579/s1, Figure S1: The SEM image, EDAX spectrum, and elemental mapping images of PB/PS; Figure S2: CV curves of (a) GCE, (b) AuNPs/PB/GCE, and (c) AuNPs/PB/PS/GCE in [Fe(CN)_6_]^3−/4−^ solution; Figure S3: The CV cycles for electrodeposition of PB; Figure S4: The CV cycles for electrodeposition of AuNPs. (a) 1 cycle, (b) 5 cycles, (c) 10 cycles, (d) 15 cycles, (e) 20 cycles; Table S1: The element contents for the obtained AuNPs/PB/PS product; Table S2: Values of the equivalent circuit parameters of the fitting curves for the different stages of the aptamer sensor preparation.
