# Supplementary material for: A Label-Free Electrochemical Aptamer Sensor for Sensitive Detection of Cardiac Troponin I Based on AuNPs/PB/PS/GCE
Source: Nanomaterials (Basel). 2024 Sep 30;14(19):1579. doi: 10.3390/nano14191579 (PMC11477680; doi:10.3390/nano14191579)
Supplement: Supplementary file 1 [file nanomaterials-14-01579-s001.zip › nanomaterials-3219516-supplementary.pdf]

Supporting Information

A Label-Free Electrochemical Aptamer Sensor for Sensitive Detection of Cardiac Troponin I Based on AuNPs/PB/PS/GCE

Liying Jiang [1], Dongyang Li [1], Mingxing Su [2], Yirong Qiu [2], Fenghua Chen [2], Xiaomei Qin [2], Lan Wang [2], Yanghai Gui [2], Jianbo Zhao [2], Huishi Guo [2], Xiaoyun Qin [2,*] and Zhen Zhang [3,*]

[1] School of Electrical and Information Engineering, Zhengzhou University of Light Industry, Zhengzhou 450000, China; jiangliying@zzuli.edu.cn (L.J.); 332201060098@zzuli.edu.cn (D.L.)

[2] School of Material and Chemical Engineering, Zhengzhou University of Light Industry, Zhengzhou 450000, China; 542204040216@zzuli.edu.cn (M.S.); 542104040314@zzuli.edu.cn (Y.Q.); phenix@zzuli.edu.cn (F.C.); qxmqin@zzuli.edu.cn (X.Q.); wanglan@zzuli.edu.cn (L.W.); yhgui@zzuli.edu.cn (Y.G.); zhaojianbo@zzuli.edu.cn (J.Z.); guohuishi@zzuli.edu.cn (H.G.)

[3] Tianjin Key Laboratory of Molecular Optoelectronic Sciences, Department of Chemistry, School of Science, Tianjin University, Tianjin 300072, China

* Correspondence: xyqin@zzuli.edu.cn (X.Q.); zhzhen@tju.edu.cn (Z.Z.)

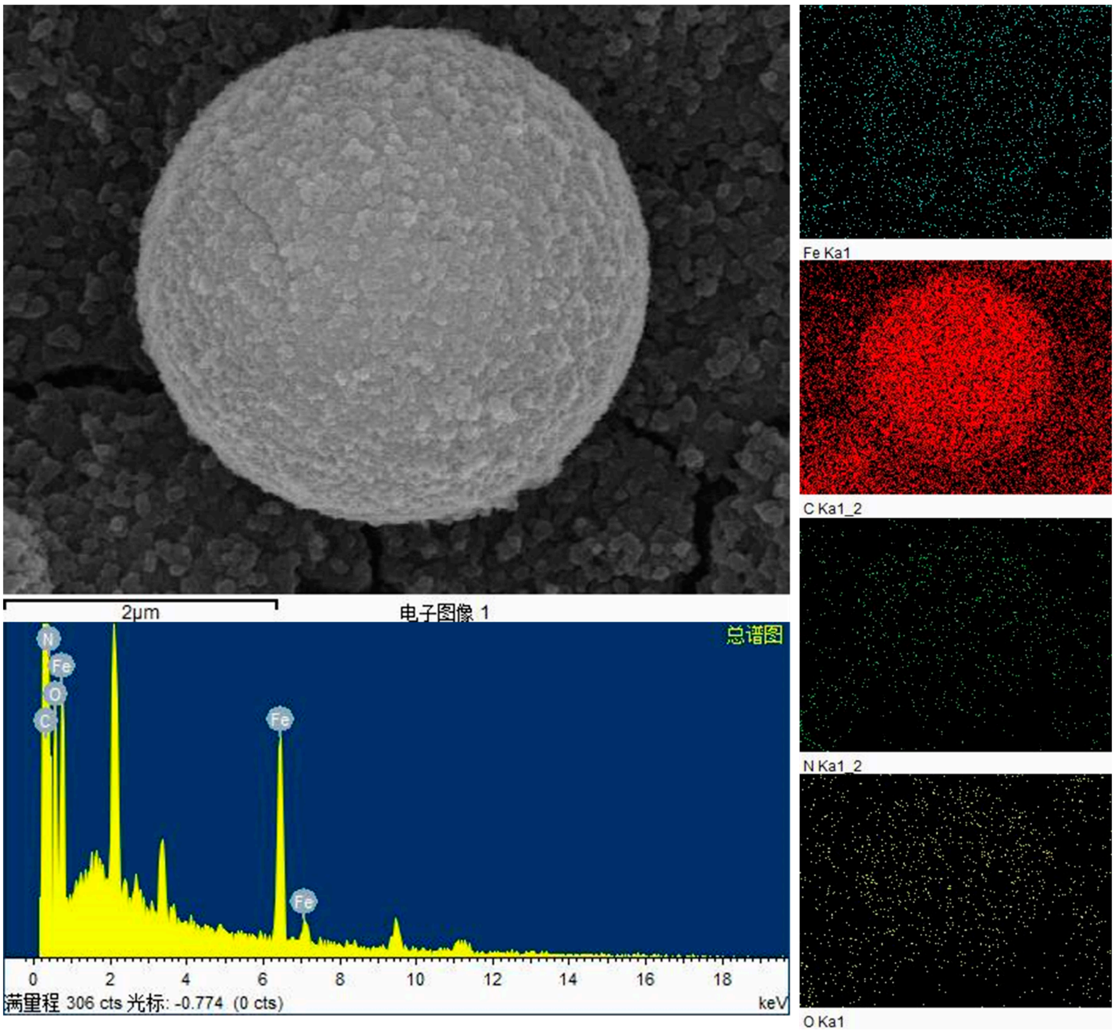

Figure S1. The SEM image, EDAX spectrum, and elemental mapping images of PB/PS.

Table S1. The element contents for the obtained AuNPs/PB/PS product.

Element	weight percentage (wt%)	atomic percentage (at%)
C	49.18	79.98
N	7.34	10.23
O	4.70	5.74
Fe	0.80	0.28
Au	37.98	3.77
Total	100	

Table S2. Values of the equivalent circuit parameters of the fitting curves for the different stages of the aptamer sensor preparation.

Electrode	R_s (ohm)	C (F)	R_{ct} (ohm)	W (S·sec^5)
Bare GCE	48.96	5.127×10^{-7}	226.6	1.52×10^{-3}
PS/GCE	51.09	6.886×10^{-6}	1202	1.39×10^{-5}
PB/PS/GCE	18.43	6.721×10^{-5}	1045	6.843×10^{-4}
AuNPs/PB/PS/GCE	2.440	3.112×10^{-8}	13.31	1.646×10^{-3}
Tro4/AuNPs/PB/PS/GCE	26.47	2.82×10^{-6}	470.5	6.952×10^{-4}
MCH/Tro4/AuNPs/PB/PS/GCE	29.88	9.402×10^{-6}	513.9	1.002×10^{-3}
cTnI/MCH/Tro4/AuNPs/PB/PS/GCE	29.68	8.541×10^{-6}	605.8	8.237×10^{-5}

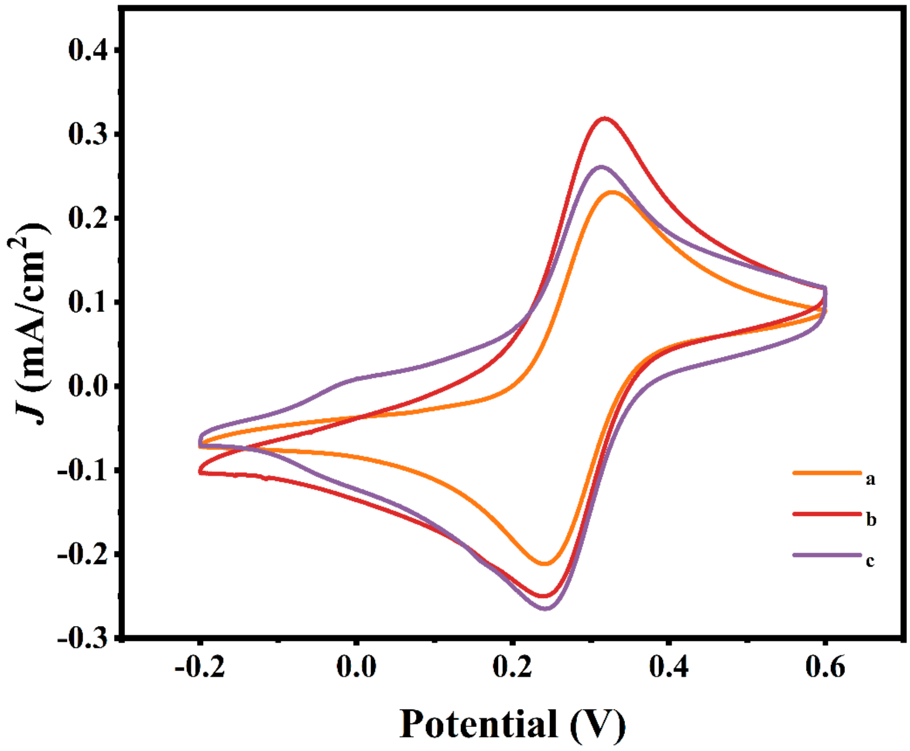

Figure S2. CV curves of (a) GCE, (b) AuNPs/PB/GCE, and (c) AuNPs/PB/PS/GCE in $[Fe(CN)_6]^{3-/4-}$ solution.

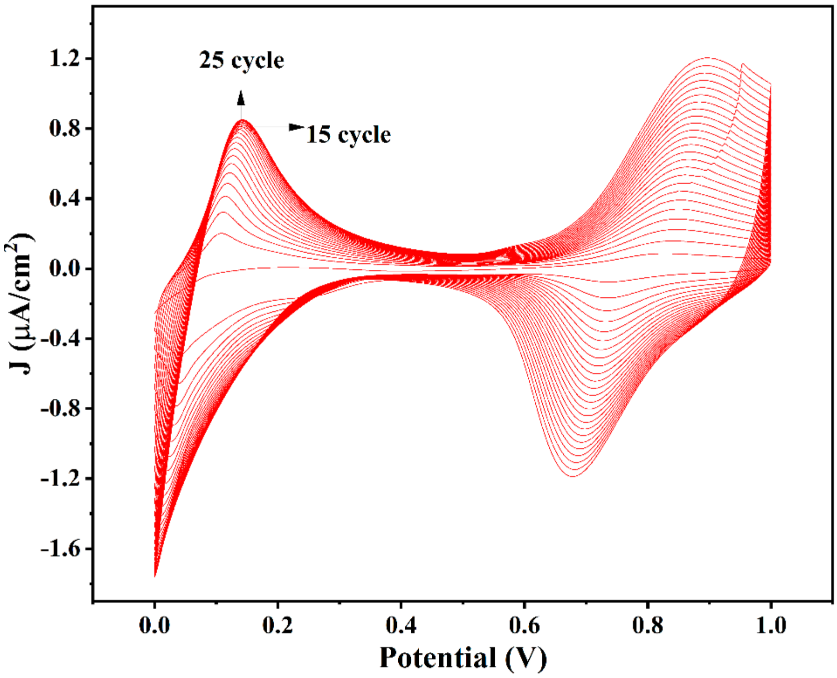

Figure S3. The CV cycles for electrodeposition of PB.

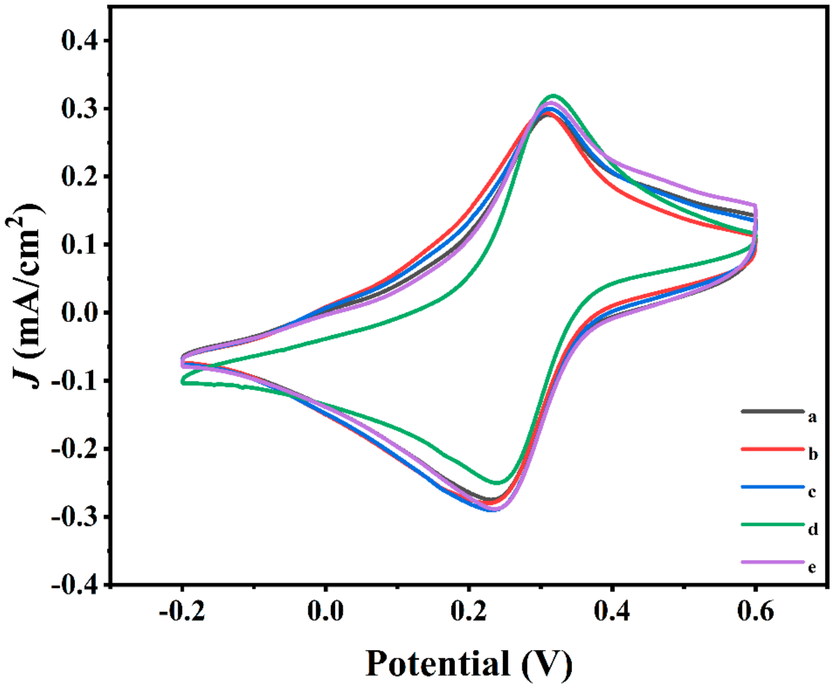

Figure S4. The CV cycles for electrodeposition of AuNPs. (a) 1 cycle, (b) 5 cycles, (c) 10 cycles, (d) 15 cycles, (e) 20 cycles.
